# Supplementary material for: Multi-Omics Profiling in Marfan Syndrome: Further Insights into the Molecular Mechanisms Involved in Aortic Disease
Source: Int J Mol Sci. 2021 Dec 31;23(1):438. doi: 10.3390/ijms23010438 (PMC8745050; doi:10.3390/ijms23010438)
Supplement: Supplementary file 1 [file ijms-23-00438-s001.zip › supplementary-material-MFS-multiomics_v27-12-2021.pdf]

SUPPLEMENTARY MATERIAL

Multi-omics profiling in Marfan syndrome: further insights into the molecular mechanisms involved in aortic disease

Judith M.A. Verhagen, Joyce Burger, Jos A. Bekkers, Alexander T. den Dekker, Jan H. von der Thüsen, Marina Zajec, Hennie T. Brüggenwirth, Marianne L.T. van der Sterre, Myrthe van den Born, Theo M. Luider, Wilfred F.J. van IJcken, Marja W. Wessels, Jeroen Essers, Jolien W. Roos-Hesselink, Robert M.W. Hofstra, Ingrid van der Pluijm, Ingrid M.B.H. van de Laar and Erwin Brosens

METHODS

Patients were offered standard genetic counseling and diagnostic testing, including SNP array analysis, whole exome sequencing and/or targeted analysis of the *FBN1* gene, depending on individual characteristics and family history.

Genome-wide SNP genotyping

Genomic DNA was extracted from whole blood samples. Copy number variation analysis was performed using HumanCytoSNP-12 v2.1 or Infinium CytoSNP-850K v1.1 BeadChips (Illumina) according to the manufacturer's protocols. Normalized output was generated with Genome Studio (Illumina) and visualized using Nexus Copy Number software (BioDiscovery).

Whole exome capture and sequencing

Genomic DNA was fragmented by sonication (Covaris), enriched for exonic sequences using the Agilent SureSelect Clinical Research Exome capture and subsequently sequenced on an Illumina HiSeq2500 with a paired-end 2x101bp protocol. For each library, at least 50 million high quality reads were obtained with a mapped fraction >0.98. The average coverage was 50x, with over 80% of the exome covered at least 20x. The resulting sequence reads were mapped against the human reference genome GRCh37/hg19 with the Burrows-Wheeler Aligner [1]. Variant calling was performed using the Genome Analysis Toolkit [2]. Cartagenia Bench Lab software was used for filtering and annotating sequence variants. We applied a virtual gene panel of 34 known HTAD related genes. Classification followed the Dutch Society of Clinical Genetic Diagnostic Laboratories practice guideline on variant interpretation. Variants were classified as pathogenic

(class 5), likely pathogenic (class 4), unknown or uncertain pathogenicity (class 3), likely benign (class 2) and benign (class 1).

Vascular smooth muscle cell culture

Vascular smooth muscle cells (VSMCs) were isolated from the ascending aorta of TAA patients and controls as described previously [3], with minor modifications. In brief, the intima and adventitia were removed, and the VSMC-rich medial layer was cut in small pieces or enzymatically dispersed with 1% collagenase type II (Worthington). VSMCs were cultured on gelatin-coated plates using the Smooth Muscle Cell Growth Medium-2 BulletKit (containing SmBM Basal Medium and SmGM-2 SingleQuots supplements, Lonza #CC-3182) at 37°C in a 3% CO_2 humidified atmosphere. Medium was changed twice a week, and cells were transferred into T25 flasks. At passage 3, VSMCs were characterized using immunofluorescent staining with anti-SMA (1:1000) and anti-SM22 alpha (1:400) antibodies, and stored at -80°C.

SUPPLEMENTARY FIGURES

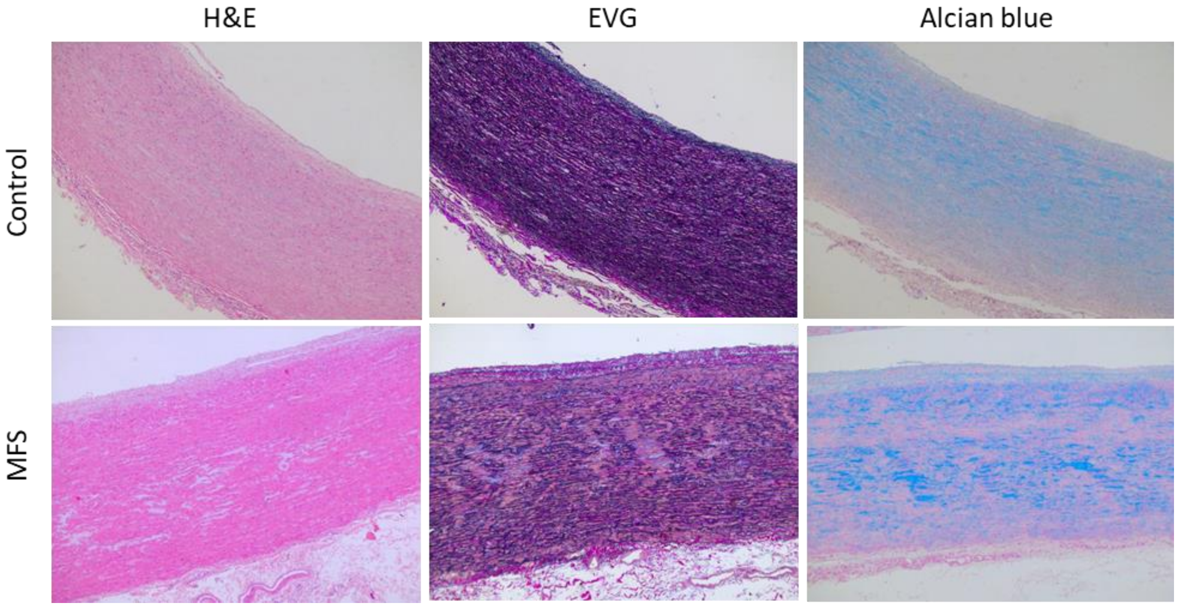

Figure S1. Representative images of histologic examination of the aortic wall from control and MFS patient. Hematoxylin and eosin (H&E), Elastica van Gieson (EVG) and alcian blue stain showing smooth muscle nuclei loss in the medial layer, fragmentation of elastic fibers (purple) and increased deposition of proteoglycans (blue) in MFS patient.

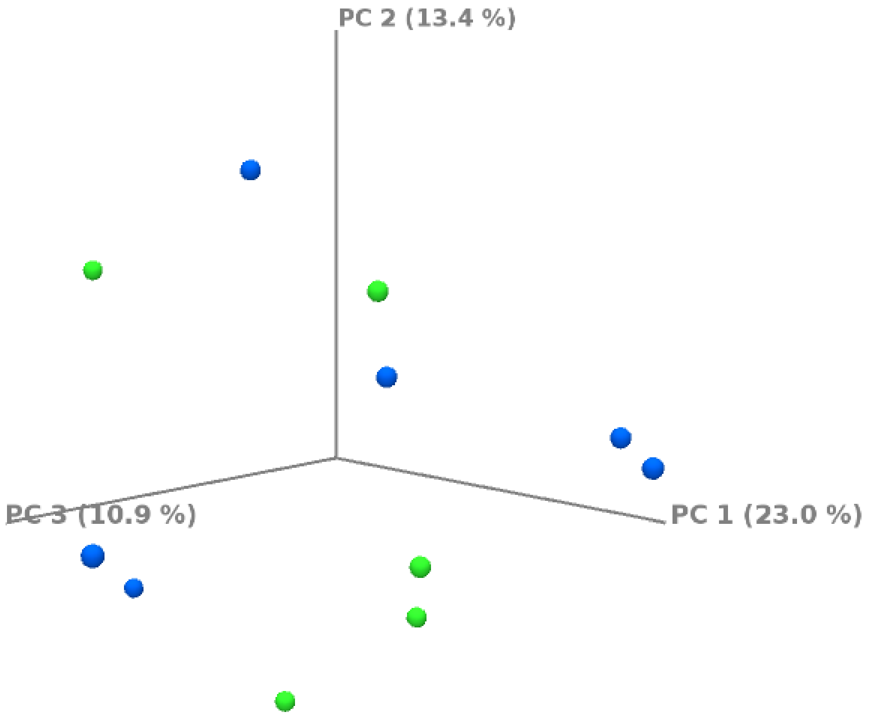

Figure S2. Three-dimensional scatter plot of principal component analysis (PCA) on gene expression data. Colors represent different genders: male = blue, female = green. The numbers in brackets correspond to the proportion of variance explained by the respective principal component. The PCA does not show clear separation of the groups.

REFERENCES

1. Li, H., and Durbin, R. (2009). Fast and accurate short read alignment with Burrows-Wheeler transform. Bioinformatics 25, 1754-1760.
2. McKenna, A., Hanna, M., Banks, E., Sivachenko, A., Cibulskis, K., Kernytsky, A., Garimella, K., Altshuler, D., Gabriel, S., Daly, M., et al. (2010). The Genome Analysis Toolkit: a MapReduce framework for analyzing next-generation DNA sequencing data. Genome Res 20, 1297-1303.
3. Proudfoot, D., and Shanahan, C. (2012). Human Vascular Smooth Muscle Cell Culture. In Human Cell Culture Protocols, R. Mitry and R. Hughes, eds. (Totowa, NJ, Humana Press), pp 251-263.
